# Updated taxonomy of Chinese *Cantharellus* subgenera *Afrocantharellus* and *Magni* (Hydnaceae, Cantharellales): Three new taxa and amended descriptions of one previous species

**DOI:** 10.3389/fmicb.2023.1109831

**Published:** 2023-03-17

**Authors:** Yu-Zhuo Zhang, Hua-Zhi Qin, Zuo-Hong Chen, Wen-Fei Lin, Zhi-Qun Liang, Shuai Jiang, Nian-Kai Zeng

**Affiliations:** ^1^Key Laboratory of Tropical Translational Medicine of Ministry of Education, School of Pharmacy, Hainan Medical University, Haikou, China; ^2^College of Science, Hainan University, Haikou, China; ^3^College of Life Science, Hunan Normal University, Changsha, China; ^4^Institute of Edible and Medicinal Fungi, College of Life Sciences, Zhejiang University, Hangzhou, China; ^5^School of Pharmaceutical Sciences and Yunnan Key Laboratory of Pharmacology for Natural Products, Kunming Medical University, Kunming, China; ^6^Yinggeling Substation, Hainan Tropical Rainforest National Park, Baisha, China

**Keywords:** chanterelle, molecular phylogeny, morphology, new taxa, taxonomy

## Abstract

*Cantharellus*, one of the main genera of Hydnaceae (Cantharellales), is both ecologically and economically important. Although many studies have focused on this genus in China, the taxonomy should be further updated. In the present study, *Cantharellus* subgenera *Afrocantharellus* and *Magni* were investigated based on morphology and molecular phylogenetic analyses with new collections from China. Five phylogenetic species were recognized among the studied collections, three of which were described as new: *C. bellus*, *C*. *cineraceus*, and *C. laevigatus*; one was previously described taxon: *C. hygrophoroides*; and the remaining species was not defined due to the paucity of the materials. Among the four described species, both *C. bellus* and *C. laevigatus* are members of subgen. *Magni*, whereas *C. cineraceus* and *C. hygrophoroides* belong to subgen. *Afrocantharellus.*

## Introduction

*Cantharellus* Adans. ex Fr. (Hydnaceae, Cantharellales) is interesting and important in forestry for its mycorrhizal properties, medicinal values, and edibility of many species (Pilz et al., 2003; Yun and Hall, 2004; Zhang M. et al., 2022). Recently, the genus has been divided into seven subgenera: *Afrocantharellus* Eyssart. & Buyck, *Cantharellus* Adans. ex Fr., *Cinnabarini* Buyck & V. Hofst., *Magni* T. Cao & H.S. Yuan, *Parvocantharellus* Eyssart. & Buyck, *Pseudocantharellus* Eyssart. & Buyck, and *Rubrini* Eyssart. & Buyck (Buyck et al., 2014; Cao et al., 2021). In China, researchers paid much attention to subgenera *Cantharellus*, *Cinnabarini*, and *Parvocantharellus* (An et al., 2017; Zhang M. et al., 2021; Zhang Y. Z. et al., 2021; Zhang M. et al., 2022; Zhang Y. Z. et al., 2022), whereas subgenera *Afrocantharellus* and *Magni* received little attention.

Subgen. *Afrocantharellus*, typified by *C. symoensii* Heinem., was proposed in 2001 (Eyssartier and Buyck, 2001), and then, Tibuhwa et al. (2012) elevated the subgenus to genus rank. However, further molecular-based infrageneric classification of *Cantharellus* did not support subgen. *Afrocantharellus* as a separate genus (Buyck et al., 2013, 2014), and thus, the subgenus rank of *Afrocantharellus* was redefined, which includes two sections, viz., *Afrocantharellus* Buyck & V. Hofstetter and *Cutirellus* Corner. It is characterized by a high proportion of four-spored basidia, an absence of clamp connections, and thin-walled hyphal extremities at the cap surface (Buyck et al., 2014). Subgen. *Magni*, a monotypic subgenus typified by *C. magnus* T. Cao & H. S. Yuan, was erected in 2021, which is characterized by a large basidioma, smooth, azonate, deep yellow to deep orange pileal surface, almost perfectly smooth hymenophore, thin- to slightly thick-walled terminal cells of pileipellis hyphae, and a presence of clamp connections (Cao et al., 2021).

In China, only two taxa of subgen. *Afrocantharellus*, viz., *C. cerinoalbus* Eyssart. & Walleyn and *C. hygrophoroides* S.C. Shao, Buyck & F.Q. Yu, and one species of subgen. *Magni*, viz., *C. magnus*, have been described/reported in previous studies (Shao et al., 2014; Song et al., 2017; Zeng and Jiang, 2020; Cao et al., 2021). Recently, several specimens of subgen. *Afrocantharellus* and *Magni* were collected in China, and they were studied using morphological and molecular phylogenetic analyses, aiming to update the taxonomy of the two subgenera.

## Materials and methods

### Morphological studies

The studied specimens were collected from Hainan, Fujian, Hunan, and Zhejiang Provinces of China and deposited in the Fungal Herbarium of Hainan Medical University (FHMU), Haikou city, Hainan Province of China. Based on detailed notes and photographs taken from fresh basidiomata, we obtained the macroscopic descriptions. Color documentation of fresh materials follows Kornerup and Wanscher (1981). Observations and measurements of microscopic features were made in 5% KOH solution and stained with 1% Congo Red (Zhang Y. Z. et al., 2022). The sections of the pileipellis were taken from the pileus between the center and the margin. The number of measured basidiospores is given as n/m/p, where “n” represents the total number of basidiospores measured from “m” basidiomata of “p” collections. The dimensions of basidiospores are presented in the form (a–)b–e–c(−d), where the range b–c contains at least of 90% of the measured values (5th to 95th percentile), “a” and “d” are the extreme values, and “e” refers to the average length/width of basidiospores. “Q” refers to the length/width ratio of basidiospores and “Q_m_” refers to the average Q of basidiospores and is given with standard deviation. For basidiospore shape, Q_m =_ 1.15–1.3 describes “broadly ellipsoid,” Q_m =_ 1.3–1.6 “ellipsoid,” and Q_m =_ 1.6–2.0 “elongate” (Yang, 2005). The terms referring to the size of basidioma are based on Bas (1969).

### Molecular procedures

The total genomic DNA was extracted from collections and dried with silica gel using the Plant Genomic DNA Kit (CWBIO, Beijing, China) according to the manufacturer’s instructions. Primer pairs used for amplification were nuc 28S rDNA D1-D2 domains (28S) with LR0R/LR5 (Vilgalys and Hester, 1990; James et al., 2006) and the translation elongation factor 1-α gene (*TEF1*) with EF1-α-F/EF1-α-R (Mikheyev et al., 2006). PCR conditions followed the program of Zhang Y. Z. et al. (2021). PCR products were checked in 1% (w/v) agarose gels. The PCR amplification products were sequenced using an ABI 3730 DNA Analyzer (Guangzhou Branch of BGI, China) with the same primers. DNA sequences were compiled with SeqMan (DNASTAR Lasergene 9) or BioEdit (Hall, 1999) and then deposited in GenBank.^1^

### Dataset assembly

Forty DNA sequences (22 of 28S and 18 of *TEF1*) from 23 collections were newly generated. The GenBank accession numbers are listed in Table 1. For the concatenated dataset, the sequences of 28S and *TEF1* from new collections were aligned with sequences of taxa of subgenera *Afrocantharellus* and *Magni*, and representative species of other subgenera of *Cantharellus* from previous studies and GenBank (Table 1). *Craterellus badiogriseus* T. Cao & H.S. Yuan was chosen as out-group inferred from Cao et al. (2021). To test for phylogenetic conflict among 28S and *TEF1*, single-gene phylogenetic trees based on each of these two fragments were analyzed. A conflict was assumed to be significant if two different relationships for the same set of taxa (one being monophyletic and the other non-monophyletic) were observed in rival trees (Vadthanarat et al., 2021). The results of analyses showed that 28S and *TEF1* were not in conflict. Thus, the datasets (28S and *TEF1*) were aligned with MUSCLE v3.6 (Edgar, 2004) and concatenated using Phyutility v2.2 for further analyses (Smith and Dunn, 2008).

**Table 1:** Taxa, vouchers, locations, and GenBank accession numbers of DNA sequences used in phylogenetic analyses.

Taxon	Voucher	Locality	GenBank accession nos.		References
			28S	*TEF1*	
*Cantharellus addaiensis*	BB 98.057	Tanzania	KF294621	JX192976	Buyck et al. (2014)
*Cantharellus addaiensis*	BB 98.033	Tanzania	KF294667	JX192992	Buyck et al. (2014)
*Cantharellus* aff. *tanzanicus*	BB 06.153	Madagascar	—	JX193009	Buyck et al. (2013)
*Cantharellus* aff. *tanzanicus*	BB 06.149	Madagascar	KF294605	JX192966	Buyck et al. (2013)
*Cantharellus albidolutescens*	BB 08.057	Madagascar	KF294645	KF294752	Buyck et al. (2014)
*Cantharellus albidolutescens*	BB 08.070	Madagascar	KF294646	JX192982	Buyck et al. (2014)
*Cantharellus albus*	KUN-HKAS 107047	Yunnan, SW China	MT782542	MT776017	Jian et al. (2020)
*Cantharellus albus*	KUN-HKAS 107048	Yunnan, SW China	MT782541	MT776016	Jian et al. (2020)
*Cantharellus altipes*	BB 07.019	United States	KF294627	GQ914939	Buyck et al. (2011, 2014)
*Cantharellus altipes*	BB 07.162	United States	KF294636	GQ914945	Buyck et al. (2011, 2014)
*Cantharellus ambohitantelyensis*	BB 08.336	Madagascar	KF294656	JX192989	Buyck et al. (2013)
*Cantharellus amethysteus*	BB 07.284	Slovakia	KF294639	GQ914953	Buyck et al. (2011, 2014)
*Cantharellus amethysteus*	BB 07.309	Slovakia	KF294642	GQ914954	Buyck et al. (2011, 2014)
***Cantharellus bellus***	**N.K. Zeng2589 (FHMU2422)**	**Hainan, southern China**	**ON117825**	**ON340619**	**Present study**
*Cantharellus cascadensis*	OSC75917	United States	AY041158	—	Dunham et al. (2003)
*Cantharellus cerinoalbus*	AV 06.051	Malaysia	KF294663	—	Buyck et al. (2014)
*“Cantharellus cerinoalbus”*	GDGM53315	Hunan, central China	KY346831	—	Song et al. (2017)
*“Cantharellus cerinoalbus”*	GDGM53341	Hunan, central China	KY346832	—	Song et al. (2017)
*“Cantharellus cerinoalbus”*	GDGM53352	Hunan, central China	KY346833	—	Song et al. (2017)
*“Cantharellus cerinoalbus”*	GDGM53375	Hunan, central China	KY346834	—	Song et al. (2017)
*Cantharellus* cf. *densifolius*	BB 11.116	Madagascar	—	KJ631733	Buyck et al. (2015)
*Cantharellus* cf. *densifolius*	BB 11.105	Madagascar	—	KJ631732	Buyck et al. (2015)
*Cantharellus cibarius*	BB 07.300	Slovakia	KF294641	GQ914950	Buyck et al. (2011, 2014)
*Cantharellus cibarius*	GE 07.025	France	KF294658	GQ914949	Buyck et al. (2011, 2014)
***Cantharellus cineraceus***	**N.K. Zeng1421 (FHMU966)**	**Fujian, SE China**	**ON089297**	**OP251152**	**Present study**
***Cantharellus cineraceus***	**N.K. Zeng1423 (FHMU968)**	**Fujian, SE China**	**ON089298**	**OP251153**	**Present study**
*Cantharellus cinnabarinus*	BB 07.053	United States	KF294630	GQ914984	Buyck et al. (2014)
*Cantharellus cinnabarinus*	BB 07.001	United States	KF294624	GQ914985	Buyck et al. (2014)
*Cantharellus citrinus*	1,691	South Korea	—	MW124385	Buyck et al. (2020a)
*Cantharellus citrinus*	1,715	South Korea	—	MW124388	Buyck et al. (2020a)
*Cantharellus coccolobae*	1,065/RC 11.25	Guadeloupe	KX857089	KX857021	Buyck et al. (2016b)
*Cantharellus coccolobae*	1,064/RC 14.24	Guadeloupe	KX857088	KX857020	Buyck et al. (2016b)
*Cantharellus curvatus*	1,695	South Korea	—	MW124390	Buyck et al. (2020a)
*Cantharellus cuticulatus*	DS 06.283	Malaysia	KF294662	—	Buyck et al. (2014)
*Cantharellus cyanescens*	DDT63	Tanzania	JQ976970	—	Tibuhwa et al. (2012)
*Cantharellus densifolius*	BB 98.013	Tanzania	KF294616	JX193014	Buyck et al. (2014)
*Cantharellus ferruginascens*	BB 07.283	England	KF294638	GQ914952	Buyck et al. (2014)
*“Cantharellus fistulosus”*	DDT31	Tanzania	JQ976959	—	Tibuhwa et al. (2012)
*“Cantharellus fistulosus”*	DDT43	Tanzania	JQ97695	—	Tibuhwa et al. (2012)
*Cantharellus floridulus*	UPS:Tibuhwa 1038.2005	Tanzania	KF294616	JX193014	Buyck et al. (2014)
*Cantharellus floridulus*	DDT33	Tanzania	JQ976962	—	Tibuhwa et al. (2012)
*Cantharellus formosus*	OSC 75930	United States	AY041164	—	Dunham et al. (2003)
*Cantharellus formosus*	OSC 76054	United States	AY041165	—	Dunham et al. (2003)
*Cantharellus goossensiae*	BB 16.063	Central African Republic	MK422953	MK422932	Buyck et al. (2020b)
*Cantharellus goossensiae*	BB 16.064	Central African Republic	MK422949	MK422928	Buyck et al. (2020b)
*Cantharellus heinemannianus*	BB 96.307	Zambia	KF294665	—	Buyck et al. (2014)
*Cantharellus humidicolus*	BB 98.0362	Tanzania	—	JX193006	Buyck et al. (2013)
*Cantharellus humidicolus*	BB 98.036	Tanzania	KF294666	JX193005	Buyck et al. (2014)
*Cantharellus hygrophoroides*	HKAS80614	Yunnan, SW China	KJ004002	KJ004003	Shao et al. (2014)
***Cantharellus hygrophoroides***	**N.K. Zeng2409 (FHMU2417)**	**Hainan, southern China**	**ON102892**	**ON237707**	**Present study**
***Cantharellus hygrophoroides***	**N.K. Zeng2493 (FHMU2420)**	**Hainan, southern China**	**ON102895**	**ON191964**	**Present study**
***Cantharellus hygrophoroides***	**N.K. Zeng2516 (FHMU1635)**	**Hainan, southern China**	**ON102891**	**ON237706**	**Present study**
***Cantharellus hygrophoroides***	**N.K. Zeng3425 (FHMU3126)**	**Hainan, southern China**	**ON102890**	**ON202824**	**Present study**
***Cantharellus hygrophoroides***	**N.K. Zeng2530 (FHMU2421)**	**Hainan, southern China**	**ON102893**	**ON237708**	**Present study**
***Cantharellus hygrophoroides***	**N.K. Zeng1579 (FHMU1058)**	**Hainan, southern China**	**ON102896**	**ON191965**	**Present study**
***Cantharellus hygrophoroides***	**S. Jiang90 (FHMU4601)**	**Hainan, southern China**	**ON102901**	—	**Present study**
***Cantharellus hygrophoroides***	**N.K. Zeng1255 (FHMU813)**	**Hainan, southern China**	**ON102899**	**ON191967**	**Present study**
***Cantharellus hygrophoroides***	**N.K. Zeng2906 (FHMU1878)**	**Hainan, southern China**	**ON102897**	**ON191966**	**Present study**
***Cantharellus hygrophoroides***	**N.K. Zeng1038 (FHMU2405)**	**Hainan, southern China**	**ON102900**	—	**Present study**
***Cantharellus hygrophoroides***	**N.K. Zeng1239 (FHMU798)**	**Hainan, southern China**	**ON102898**	—	**Present study**
***Cantharellus hygrophoroides***	**N.K. Zeng481 (FHMU2397)**	**Hainan, southern China**	**ON102894**	—	**Present study**
***Cantharellus hygrophoroides***	**N.K. Zeng483 (FHMU2398)**	**Hainan, southern China**	**ON102902**	**ON191968**	**Present study**
*Cantharellus ibityensis*	BB 08.203	Madagascar	KF294651	JX192985	Buyck et al. (2014)
*Cantharellus ibityensis*	BB 08.196	Madagascar	KF294650	GQ914980	Buyck et al. (2014)
*Cantharellus koreanus*	697/V. Antonin 14.115	Korea	—	KY271940	Antonín et al. (2017)
*Cantharellus koreanus*	1,689/V. Antonin 13.136	Korea	—	KY271941	Antonín et al. (2017)
***Cantharellus laevigatus***	**Z.H. Chen MHHNU32009 (FHMU6950)**	**Hunan, central China**	**ON117819**	**ON340612**	**Present study**
***Cantharellus laevigatus***	**Z.H. Chen MHHNU32014 (FHMU6952)**	**Hunan, central China**	**ON117820**	**ON340613**	**Present study**
***Cantharellus laevigatus***	**Z.H. Chen MHHNU32011 (FHMU6953)**	**Hunan, central China**	**ON117823**	**ON340616**	**Present study**
***Cantharellus laevigatus***	**Z.H. Chen MHHNU32061 (FHMU6954)**	**Hunan, central China**	**ON117818**	**ON340611**	**Present study**
***Cantharellus laevigatus***	**Z.H. Chen MHHNU32013 (FHMU6955)**	**Hunan, central China**	**ON117824**	**ON340615**	**Present study**
***Cantharellus laevigatus***	**W.F. Lin4-2 (FHMU6956)**	**Zhejiang, eastern China**	**ON117821**	**ON340614**	**Present study**
*Cantharellus lateritius*	BB 07.004	United States	—	GQ914955	Buyck et al. (2011)
*Cantharellus lateritius*	BB 07.025	United States	KF294628	GQ914957	Buyck et al. (2011, 2014)
*Cantharellus luteostipitatus*	BB 11.045	Madagascar	—	KP033511	Liu et al. (2015)
*Cantharellus luteostipitatus*	BB 11.044	Madagascar	—	KP033510	Liu et al. (2015)
*Cantharellus luteostipitatus*	BB 11.042	Madagascar	—	KP033509	Liu et al. (2015)
*Cantharellus macrocarpus*	N.K. Zeng4050 (FHMU3304)	Hainan, southern China	MT986061	MT990634	Zhang Y. Z. et al. (2021)
*Cantharellus macrocarpus*	N.K. Zeng4207 (FHMU3357)	Hainan, southern China	MT986063	—	Zhang Y. Z. et al. (2021)
*Cantharellus magnus*	Wei 10,319	Liaoning, NE China	MW979516	MW999421	Cao et al. (2021)
*Cantharellus magnus*	Wei 10,244	Liaoning, NE China	MW979517	MW999420	Cao et al. (2021)
*Cantharellus miniatescens*	1,683/TH9870	Cameroon	KX857108	KX857079	Buyck et al. (2016a)
*Cantharellus minor*	BB 07.057	United States	KF294632	JX192979	Buyck et al. (2014)
*Cantharellus minor*	BB 07.002	United States	KF294625	JX192978	Buyck et al. (2014)
*Cantharellus parvisporus*	BB 98.020	Tanzania	KF294614	JX192972	Buyck et al. (2014)
*Cantharellus parvisporus*	BB 98.037	Tanzania	KF294611	GQ914966	Buyck et al. (2014)
*Cantharellus phasmatis*	C057	United States	JX030431	JX030417	Foltz et al. (2013)
*Cantharellus phasmatis*	C074	United States	—	JX030418	Foltz et al. (2013)
*Cantharellus phloginus*	HKAS 58209	China	KF801101	KF801096	Shao et al. (2016a)
*Cantharellus phloginus*	HKAS 58208	China	KF801100	KF801095	Shao et al. (2016a)
*Cantharellus platyphyllus*	BB 98.126	Tanzania	KF294620	JX192975	Buyck et al. (2014)
*Cantharellus platyphyllus*	BB 98.012	Tanzania	KF294617	GQ914969	Buyck et al. (2014)
*Cantharellus platyphyllus*	DDT78	Tanzania	JQ976978	—	Tibuhwa et al. (2012)
*Cantharellus platyphyllus*	DDT03	Tanzania	JQ976950	—	Tibuhwa et al. (2012)
*Cantharellus platyphyllus*	DDT41	Tanzania	JQ976964	—	Tibuhwa et al. (2012)
*Cantharellus platyphyllus* subsp. *bojeriensis*	BB 08.160	Madagascar	KF294648	JX192984	Buyck et al. (2014)
*Cantharellus platyphyllus* subsp. *bojeriensis*	BB 08.158	Madagascar	KF294647	JX192983	Buyck et al. (2014)
*Cantharellus pseudominimus*	JV 00.663	Portugal	KF294657	JX192991	Buyck et al. (2014)
*Cantharellus quercophilus*	BB 07.097	United States	KF294644	JX192981	Buyck et al. (2014)
*Cantharellus roseofagetorum*	AH44786	Georgia	KX828813	KX828840	Olariaga et al. (2017)
*Cantharellus roseofagetorum*	AH44789	Georgia	KX828812	KX828839	Olariaga et al. (2017)
*Cantharellus ruber*	UPS:Tibuhwa 1045.2007	Tanzania	JQ976966	—	Tibuhwa et al. (2012)
*Cantharellus sebosus*	BB 08.234	Madagascar	KF294652	JX192986	Buyck et al. (2014)
*Cantharellus sebosus*	BB 08.162	Madagascar	KF294649	GQ914981	Buyck et al. (2014)
*Cantharellus* sp.	BB 06.179	Madagascar	KF294607	JX192968	Buyck et al. (2014)
*Cantharellus splendens*	BB 96.199	Zambia	KF294671	—	Buyck et al. (2014)
*Cantharellus splendens*	BB 96.306	Zambia	KF294670	—	Buyck et al. (2014)
*Cantharellus splendens*	DDT57	Tanzania	JQ976967	—	Tibuhwa et al. (2012)
*Cantharellus splendens*	DDT17	Tanzania	JQ976956	—	Tibuhwa et al. (2012)
*Cantharellus splendens*	JD 896	Congo	—	KX834396	Kesel et al. (2016)
*Cantharellus splendens*	ADK 6071	Congo	—	KX834395	Kesel et al. (2016)
*Cantharellus splendens*	JD968	Congo	—	KX834397	Kesel et al. (2016)
*Cantharellus subincarnatus* subsp. *rubrosalmoneus*	BB 06.096	Madagascar	KF294602	JX192963	Buyck et al. (2014)
*Cantharellus subincarnatus* subsp. *rubrosalmoneus*	BB 06.080	Madagascar	KF294601	JX192962	Buyck et al. (2014)
*Cantharellus symoensii*	BB 98.113	Tanzania	KF294619	JX192974	Buyck et al. (2014)
*Cantharellus symoensii*	BB 98.011	Tanzania	KF294618	GQ914970	Buyck et al. (2014)
*Cantharellus symoensii*	DDT36	Tanzania	JQ976961	—	Tibuhwa et al. (2012)
*Cantharellus symoensii*	DDT04	Tanzania	JQ976951	—	Tibuhwa et al. (2012)
*Cantharellus symoensii*	DDT66	Tanzania	JQ976971	—	Tibuhwa et al. (2012)
*Cantharellus symoensii*	DDT11	Tanzania	JQ976953	—	Tibuhwa et al. (2012)
*Cantharellus symoensii*	DDT67	Tanzania	JQ976972	—	Tibuhwa et al. (2012)
*Cantharellus symoensii*	DDT14	Tanzania	JQ976955	—	Tibuhwa et al. (2012)
*Cantharellus tabernensis*	BB 07.020	United States	—	GQ914971	Buyck et al. (2011)
*Cantharellus tabernensis*	BB 07.056	United States	—	GQ914974	Buyck et al. (2011)
*Cantharellus texensis*	BB 07.018	United States	KF294626	GQ914988	Buyck et al. (2014)
*Cantharellus texensis*	BB 07.120	United States	JN940601	GQ914987	Buyck et al. (2014)
*Cantharellus tomentosus*	BB 98.060	Tanzania	KF294672	JX192995	Buyck et al. (2014)
*Cantharellus tomentosus*	BB 98.038	Tanzania	KF294610	GQ914965	Buyck et al. (2014)
*Cantharellus tricolor*	BB 06.247	Madagascar	—	JX193017	Buyck et al. (2013)
*Cantharellus versicolor*	KUN-HKAS 55761	Yunnan, SW China	—	KM893856	Shao et al. (2016b)
*Cantharellus versicolor*	KUN-HKAS 58242	Yunnan, SW China	—	KM893857	Shao et al. (2016b)
*Cantharellus violaceoflavescens*	ADK 4791	Togo	MT006308	—	Buyck et al. (2020b)
*Cantharellus violaceoflavescens*	ADK 4790	Togo	MT006307	—	Buyck et al. (2020b)
*Cantharellus yunnanensis*	XieXD174	Yunnan, SW China	KU720333	KU720337	Unpublished
*Cantharellus yunnanensis*	ZhangJP117	Yunnan, SW China	KU720336	—	Unpublished

GenBank numbers in bold indicate the newly generated sequences; SW, Southwestern China, SE, Southeastern China, and NE, Northeastern China.

### Phylogenetic analyses

The combined nuclear dataset (28S + *TEF1*) was analyzed using maximum likelihood (ML) and Bayesian inference (BI). For ML, tree generation and bootstrap analyses were performed with RAxML7.2.6 (Stamatakis, 2006) running 1,000 replicates combined with an ML search. For BI, the best-fit model of substitution among those implementable in MrBayes was estimated separately for each character set using jModelTest (Darriba et al., 2012) on the CIPRES portal, based on the Bayesian information criterion. The best-fit likelihood models for 28S and *TEF1* were GTR + I + G and SYM + I + G, respectively. BI was conducted in MrBayes v3.1 (Huelsenbeck and Ronquist, 2005) on the CIPRES Science Gateway portal (Miller et al., 2011). For the BI analyses, four chains were processed with the generation set as 10 million using the selected model for each gene. The trees were sampled every 100 generations. Other parameters were kept at their default setting. The chain convergence was determined using Tracer v 1.5^2^ to ensure sufficiently large ESS values. The stop rule was used when parallel MCMC runs converged (ESS value > 200). Finally, 6.5 million generations were taken to reach the convergence, and the average deviation of split frequencies was 0.009922. The trees were summarized, and statistical values were obtained using the sump and sumt commands with burn-ins (i.e., the first 25% of the samples) discarded. In addition, phylogenetic distances of some *Cantharellus* species were calculated using MEGA 11 (Tamura et al., 2021).

## Results

### Molecular data

**Figure 1:**
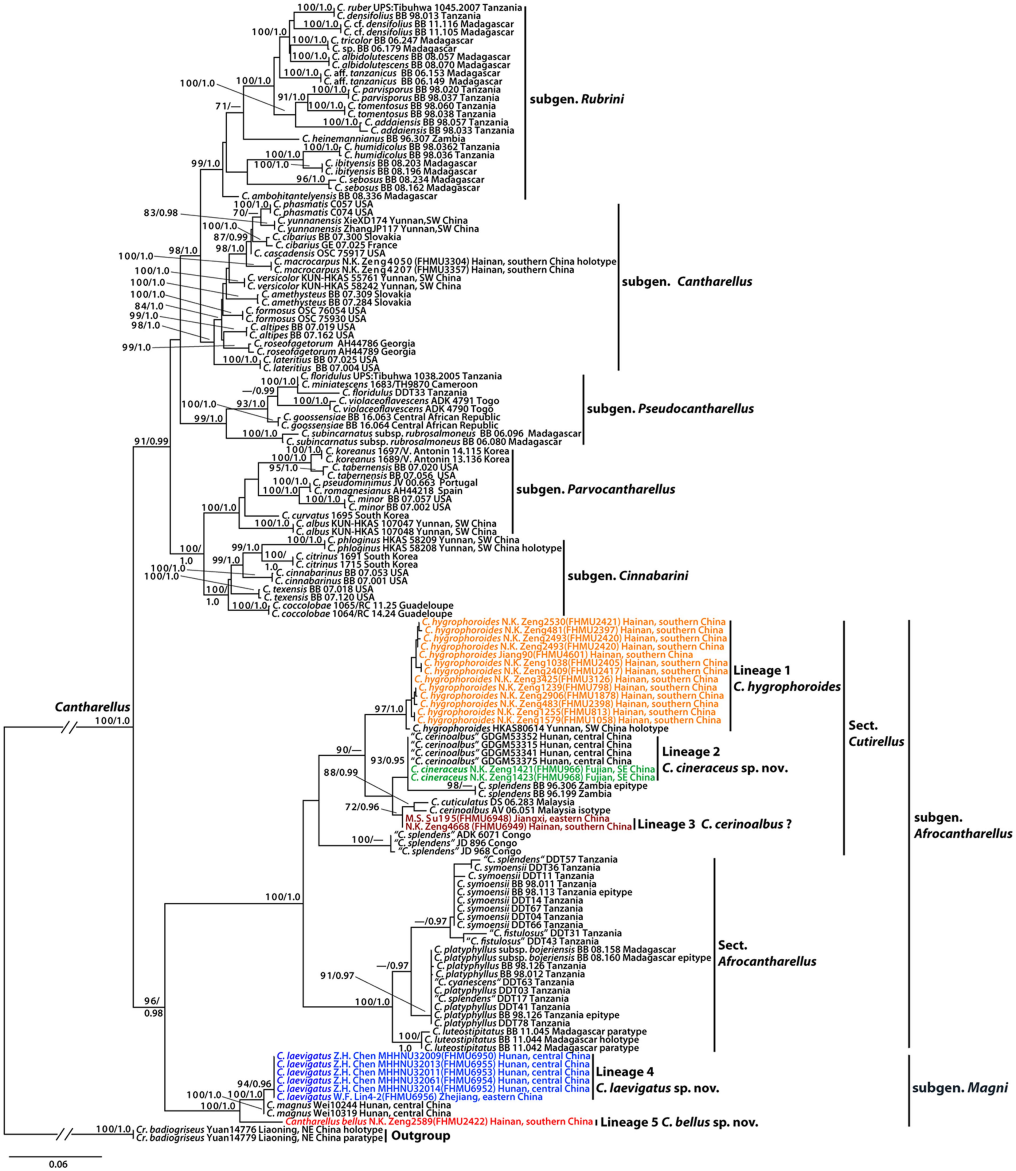
Phylogram of genus *Cantharellus*, with emphasis on subgenera *Afrocantharellus* and *Magni*, inferred from a two-locus (rDNA 28S, *TEF1*) dataset using RAxML. BS (≥70%) and PP (≥0.95) are indicated above the branches.

The combined dataset (28S + *TEF1*) consisted of 139 taxa and 1862 nucleotide sites (986 of 28S and 876 of *TEF1*). The phylogram with branch lengths generated from RAxML and support values (BS and PP) is shown in Figure 1. The topologies of phylogenetic trees generated from ML and BI analyses were identical, although statistical support for some branches showed slight differences.

The present molecular data indicated that the Chinese collections of subgenera *Afrocantharellus* and *Magni* were grouped into five independent lineages (Figure 1). Lineage 1, with strong statistical support (BS = 97%, PP = 1.0), included the holotype of *C. hygrophoroides* (HKAS80614) and 13 new collections (FHMU798, FHMU813, FHMU1058, FHMU1635, FHMU1878, FHMU2397, FHMU2398, FHMU2405, FHMU2417, FHMU2420, FHMU2421, FHMU3126, and FHMU4601) from southern China; lineage 2, with high statistical support (BS = 93%, PP = 0.95), included two new specimens (FHMU966 and FHMU968) from southeastern China and four collections labeled as *C. cerinoalbus* from central China; lineage 3, with weak statistical support, was comprised of two new specimens (FHMU6948 and FHMU6949) from eastern China and southern China, respectively; lineage 4, with strong statistical support (BS = 94%, PP = 0.96), was comprised of five new collections (FHMU6950, FHMU6952, FHMU6953, FHMU6954, and FHMU6955) from central China and one new specimen (FHMU6956) from eastern China; lineage 5, only included one new collection (FHMU2422) from southern China (Figure 1).

### Taxonomy

***Cantharellus bellus*** N.K. Zeng, Y.Z. Zhang & Zhi Q. Liang, sp. nov.

Figures 2A,B, 3.

**Figure 2:**
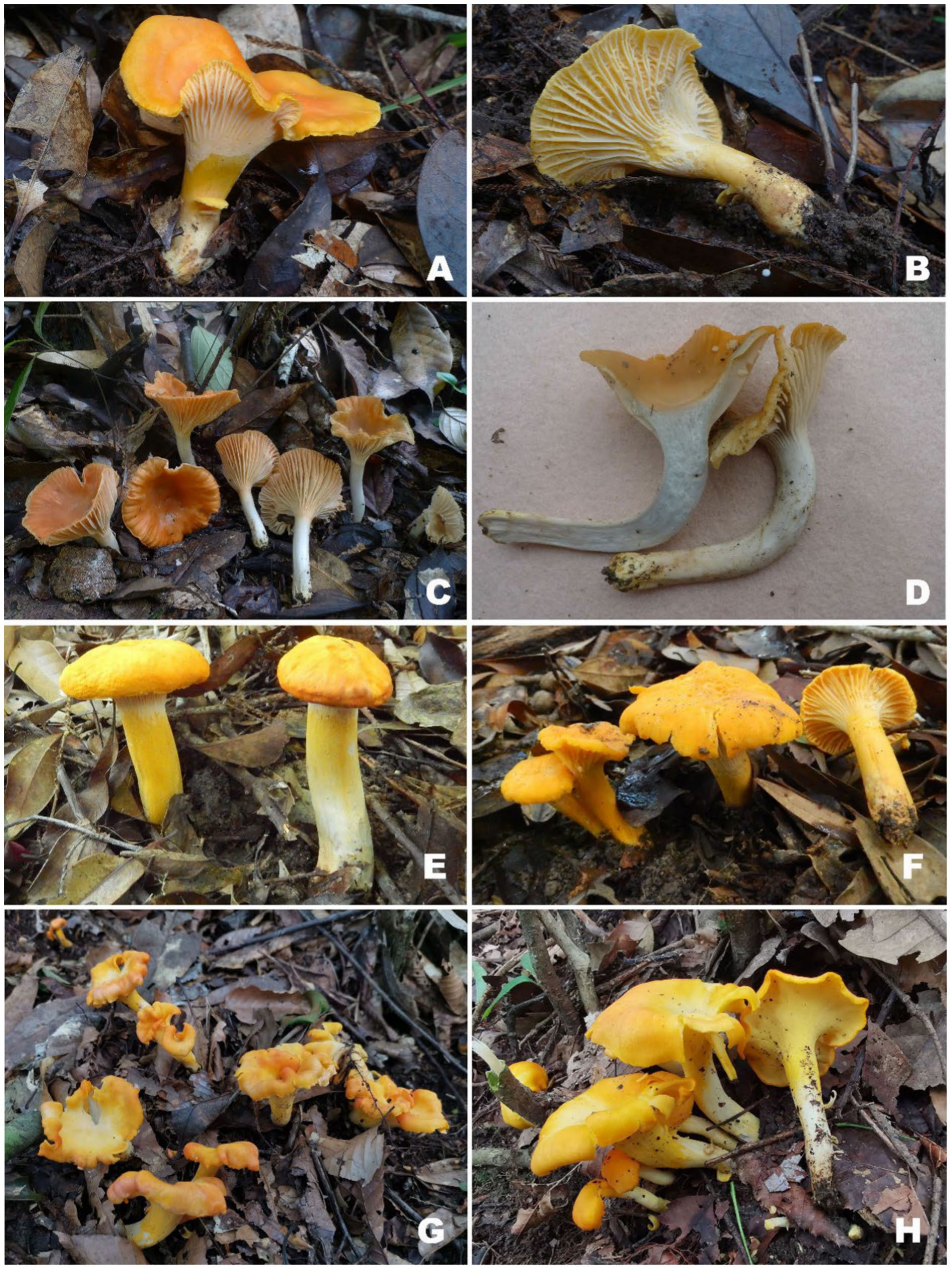
Basidiomata of *Cantharellus* subg. *Afrocantharellus* species. **(A,B)** *Cantharellus bellus* (FHMU2422, holotype); **(C,D)** *Cantharellus cineraceus* **(C)** FHMU968, holotype; **(D)** FHMU966; **(E,F)** *Cantharellus hygrophoroides* **(E)** FHMU2421; **(F)** FHMU1635; **(G,H)** *Cantharellus laevigatus* **(G)** FHMU6955, holotype; **(H)** FHMU6952; Photographs: **(A–F)** N. K. Zeng; **(G,H)** Z. H. Chen.

**Figure 3:**
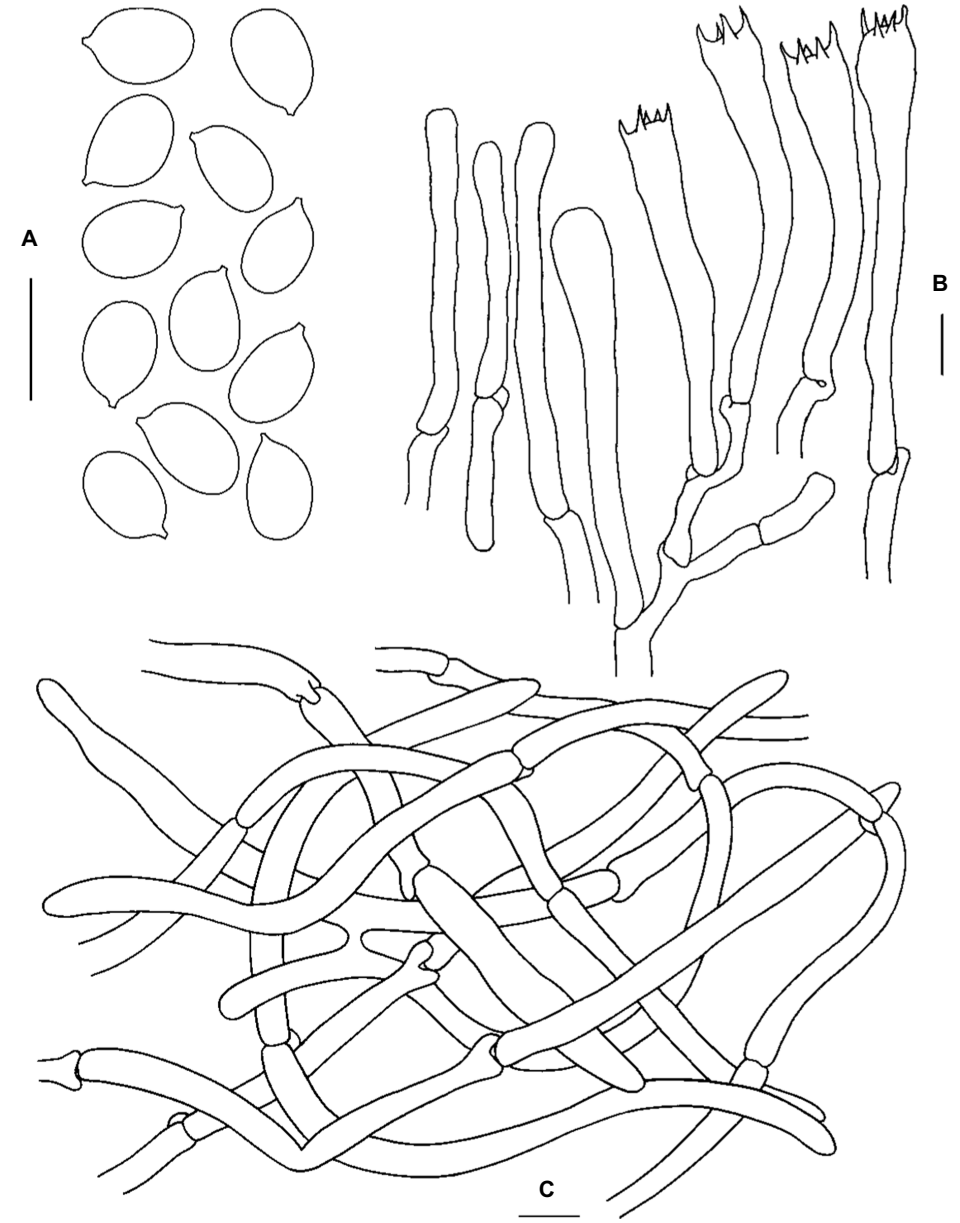
Microscopic features of *Cantharellus bellus* (FHMU2422, holotype). **(A)** Basidiospores. **(B)** Basidia. **(C)** Pileipellis. Scale bars = 10 μm. Drawings by Y. Z. Zhang.

MycoBank: MB845017.

Diagnosis: Differs from other species of *Cantharellus* subgen. *Magni* by a bright orange-yellow pileus, a well-developed hymenophore, and an intricate trichodermal pileipellis.

Etymology: The specific epithet “*bellus*” refers to the beautiful basidioma of the new species.

Holotype: China. Hainan Province: Yinggeling of Hainan Tropical Rainforest National Park, elev. 650 m, 5 August 2015, N. K. Zeng2589 (FHMU2422). GenBank accession number: 28S = ON117825, *TEF1* = ON340619.

**Basidiomata** small-sized. **Pileus** 2.5–5 cm in diameter, plano-convex to infundibuliform, margin irregularly wavy; surface smooth, bright orange-yellow (5A6), shiny; context above stipe about 0.2 cm in thickness, yellowish (3A1), unchanging in color when injured. **Hymenophore** composed of well-developed, well-spaced, decurrent, unequal and forked gill folds, commonly anastomosing; folds about 0.15 cm broad, pale yellow (3A3). **Stipe** 2.5–3.7 × 0.45–0.6 cm, subcylindrical, central, solid, hollow when old; surface dry, grayish yellow (4A2) to fulvous (2B2); context yellowish (1A2). **Taste** and **Odor** not distinctive. **Spore print** not obtained.

**Basidiospores** [40/2/1] (7–)7.5–8.7–9.5(−10) × 5.5–6.3–6.5(−7) μm, Q = (1.08–)1.15–1.55(−1.73), Q_m_ = 1.39 ± 0.12, broadly ellipsoid to ellipsoid, slightly thick-walled (up to 0.5 μm), smooth, yellowish in KOH. **Basidia** 55–83 × 8–10.5 μm, narrow, subcylindric, slightly thick-walled (0.5–0.7 μm), 3–5–spored, yellowish in KOH; sterigmata 5.5–7.5 μm in length. **Cystidia** absent. **Pileipellis** an intricate trichoderm composed of cylindrical, 4–7.5 μm wide, slightly thick-walled (up to 0.5 μm) hyphae, faintly pale yellow in KOH; terminal cells 20–100 × 4.5–7 μm, slightly thick-walled (up to 0.5 μm), subcylindrical to subclavate, with obtuse apex. **Clamp connections** present in all tissues.

Habitat: Solitary on the ground of forests dominated by *Castanopsis kawakamii* Hayata.

Known distribution: Southern China (Hainan Province).

Notes: Phylogenetically, *C. bellus* is closely related to *C. magnus* (Figure 1), also a member of subgen. *Magni*; however, the latter has a larger basidioma (pileus up to 20 cm in diameter), a smooth hymenophore, and larger basidiospores measuring (8.5–)9–11(−11.5) × (6.5–)6.8–7.5(−8.0) μm (Cao et al., 2021). *Cantharellus chuiweifanii* N.K. Zeng, Y.Z. Zhang, and Zhi Q. Liang, *C. cibarius* Fr., *C. pallens* Pilát, and *C. yunnanensis* W.F. Chiu are morphologically similar to *C. bellus*. However, the four species are all members of subgen. *Cantharellus* (Zhang Y. Z. et al., 2022).

***Cantharellus cineraceus*** N.K. Zeng, Y.Z. Zhang & Zhi Q. Liang, sp. nov.

Figures 2C,D, 4.

**Figure 4:**
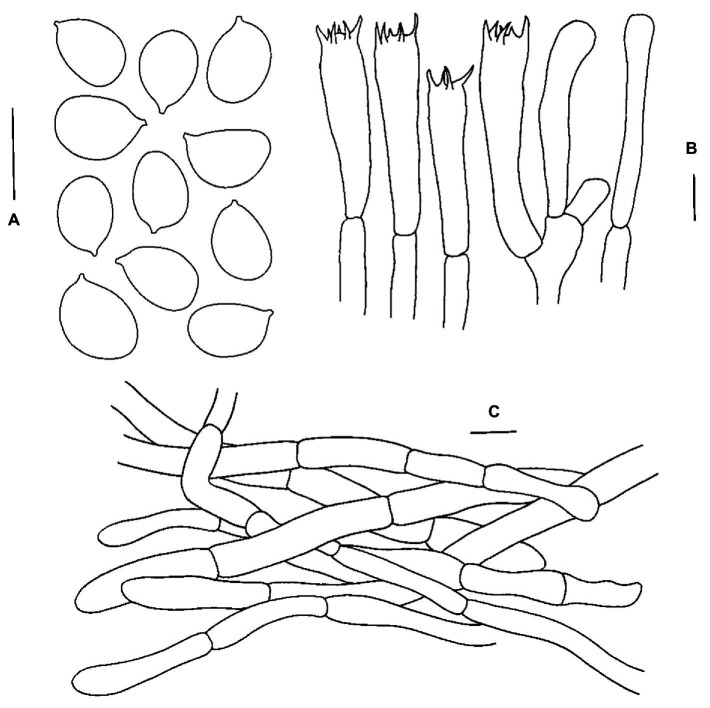
Microscopic features of *Cantharellus cineraceus* (FHMU968, holotype). **(A)** Basidiospores. **(B)** Basidia. **(C)** Pileipellis. Scale bars = 10 μm. Drawings by Y. Z. Zhang.

MycoBank: MB845018.

Diagnosis: Differs from other species of *Cantharellus* subgen. *Afrocantharellus* by a yellowish orange pileus, grayish white stipe and context, and it is associated with fagaceous trees.

Etymology: The specific epithet “*cineraceus*” refers to the grayish-white context of the new species.

Holotype: China. Fujian Province: Zhangping city, Xinqiao town, Chengkou village, elev. 350 m, 17 August 2013, N. K. Zeng1423 (FHMU968). GenBank accession number: 28S = ON089298, *TEF1* = OP251153.

**Basidiomata** small-sized. **Pileus** 3–4.5 cm in diameter, central depression to broadly infundibuliform, margin slightly incurved, irregular, often wavy and lobed; surface smooth, yellowish orange (6B6); context above stipe 0.2–0.3 cm in thickness, yellowish (4A2), unchanging in color when injured. **Hymenophore** composed of well-developed, well-spaced, decurrent, unequal, occasionally to commonly forked gill folds, mostly anastomosing near the cap margin; folds 0.05–0.1 cm broad, white (2A1), pale orange-ochre (4A3) to pale ochre-yellow (2A4). **Stipe** 2.5–3.5 × 0.5–0.8 cm, subcylindrical, central, solid, hollow when old; surface dry, grayish white (2A1); context grayish white (3A1). **Taste** and **Odor** not distinctive. **Spore print** not obtained.

**Basidiospores** [79/9/2] 8–9.3–10(−11.5) × (5–)5.5–6.4–7 μm, Q = 1.23–1.73(−1.9), Q_m_ = 1.46 ± 0.14, broadly ellipsoid to ellipsoid, slightly thick-walled (up to 0.5 μm), smooth, yellowish in KOH. **Basidia** 56–98 × 8–11 μm, long, narrow, subcylindric, slightly thick-walled (up to 0.5 μm), 3–6–spored, yellowish in KOH; sterigmata 6–8 μm in length. **Cystidia** absent. **Pileipellis** a cutis composed of cylindrical, 5.5–10 μm wide, slightly thick-walled (0.5–0.8 μm) hyphae, faintly pale yellow in KOH; terminal cells 24–36 × 5–8.5 μm, thin- to slightly thick-walled (up to 0.5 μm), subcylindrical to subclavate, with obtuse apex. **Clamp connections** absent in all tissues.

Habitat: Gregarious on the ground in forests dominated by *Castanopsis kawakamii* Hayata.

Known distribution: Southeastern and central China (Fujian and Hunan Provinces).

Additional specimen examined: China. Fujian Province: Zhangping city, Xinqiao town Chengkou village, elev. 350 m, 17 August 2013, N. K. Zeng1421 (FHMU966).

Notes: Although the values of phylogenetic distances among *C. cineraceus*, *C. cerinoalbus* Eyssart. & Walleyn, and *C. cuticulatus* Corner seem to be not high (Figure 1) for only 28S sequences of the three taxa were obtained, *C. cerinoalbus* and *C. cuticulatus* are morphologically different with *C. cineraceus*. Malaysian *C. cerinoalbus* has a yellow cap often with olivaceous or grayish tinges, smaller basidiospores measuring 7.5–9(−10) × 5–5.75(−6) μm, and it is associated with trees of Dipterocarpaceae (Eyssartier et al., 2009); *C. cuticulatus*, originally described from Malaysia, has a larger basidioma (pileus up to 7.5 cm in diameter), a stipe tinged with yellow, and a trichodermal structure of the pileipellis (Corner, 1966; Buyck et al., 2014). In future, more interesting information will be provided with more collections made and more genes investigated.

*Cantharellus splendens* Buyck is also morphologically similar to *C. cineraceus*. However, the former has narrower basidiospores measuring 8–9.5–11 × 5–5.25–6 μm, a trichodermal structure of the pileipellis, and it is distributed in Africa (Buyck, 2012; Buyck et al., 2014).

***Cantharellus hygrophoroides*** S.C. Shao, Buyck & F.Q. Yu, Cryptog. Mycol. 35(3): 287, 2014.

Figures 2E,F, 5.

**Figure 5:**
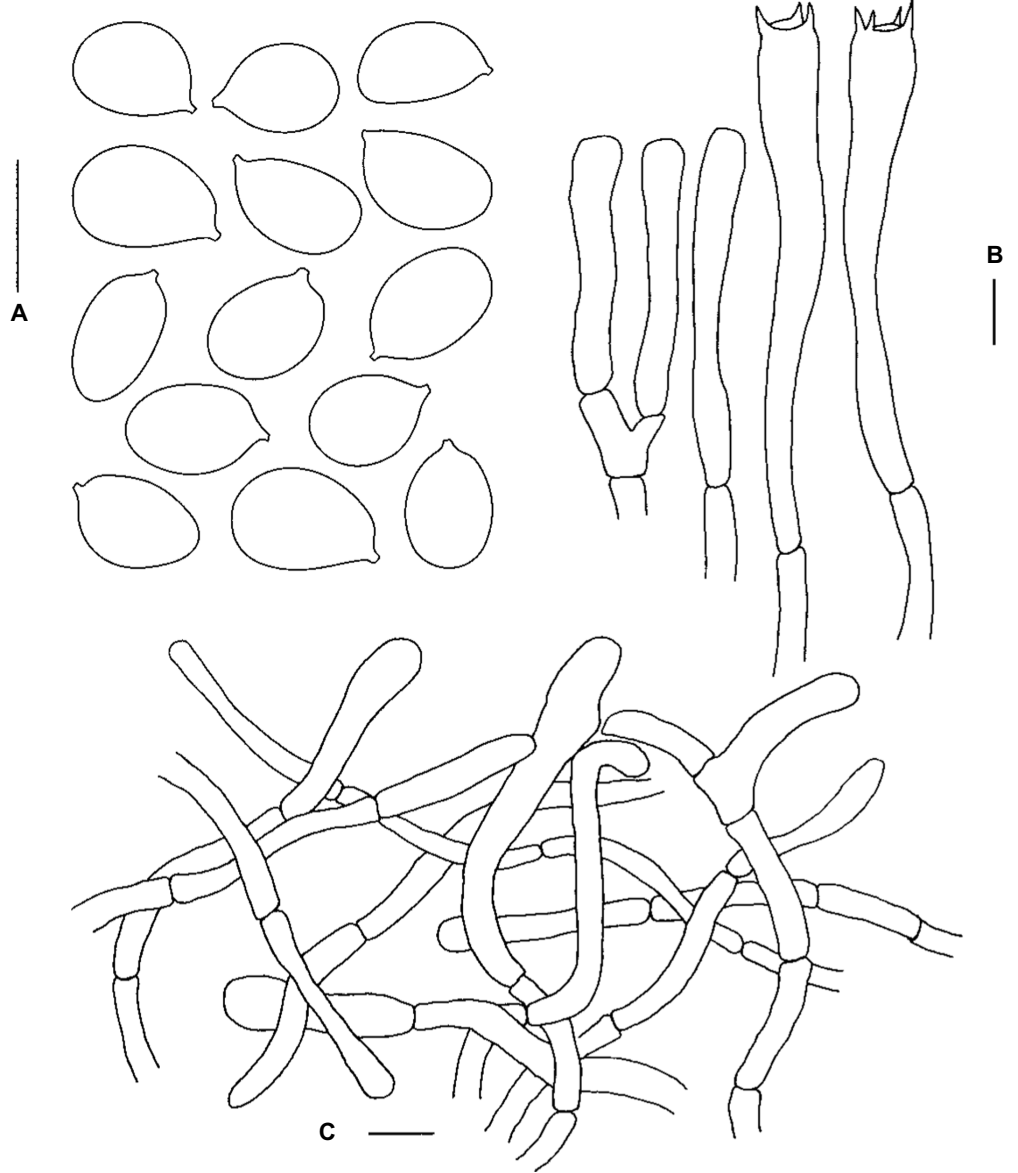
Microscopic features of *Cantharellus hygrophoroides* (FHMU1635). **(A)** Basidiospores. **(B)** Basidia. **(C)** Pileipellis. Scale bars = 10 μm. Drawings by Y. Z. Zhang.

**Basidiomata** medium-sized. **Pileus** 5–12 cm in diameter, convex with central depression at first, becoming infundibuliform at maturity, margin inrolled and slight lobed; surface smooth, orange-yellow (3A6); context above stipe 0.3 cm in thickness, white (3A1), unchanging in color when injured. **Hymenophore** composed of well-developed, well-spaced, decurrent, unequal, occasionally to commonly forked gill folds, mostly anastomosing near the cap margin; folds about 0.5 cm broad, pale yellow (3A4). **Stipe** 4.5–9 × 0.9–1.5 cm, subcylindrical, central, solid, hollow when old; surface dry, lemon yellow (1A5) to yellowish (2A1). **Taste** and **Odor** not distinctive. **Spore print** not obtained.

**Basidiospores** [254/18/13] (8–)9–9.92–11(−12) × (5.5–)6–6.87–8(−9) μm, Q = (1.19–)1.25–1.67(−1.83), Q_m_ = 1.45 ± 0.12, broadly ellipsoid to ellipsoid to elongate, slightly thick-walled (up to 0.5 μm), smooth, yellowish in KOH. **Basidia** 65–82 × 9–12 μm, narrowly clavate, thin-walled to slightly thick-walled (0.4–0.5 μm), 3–5–spored, yellowish in KOH; sterigmata 3.5–5 μm in length. **Cystidia** absent. **Pileipellis** an intricate trichoderm composed of cylindrical, 4–9 μm wide, slightly thick-walled (0.5–0.8 μm) hyphae, faintly pale yellow in KOH; terminal cells 17–54 × 3.5–8.5 μm, thin- to slightly thick-walled (up to 0.5 μm), subcylindrical to subclavate, with obtuse apex. **Clamp connections** absent in all tissues.

Habitat: Solitary, scattered, or gregarious on the ground of forests dominated by fagaceous trees such as *Castanopsis fissa* (Champion ex Bentham) Rehder et E. H. Wilson.

Known distribution: Southwestern China (Yunnan Province; Shao et al., 2014) and southern China (Hainan Province).

Specimens examined: China. Hainan Province: Yinggeling of Hainan Tropical Rainforest National Park, elev. 650 m, 17 June 2013, N. K. Zeng1239 (FHMU798); same location, elev. 650 m, 31 July 2015, N. K. Zeng2409 (FHMU2417); same location, elev. 650 m, 3 August 2015, N. K. Zeng2493 (FHMU2420); same location, elev. 650 m, 4 August 2015, N. K. Zeng2530 (FHMU2421); same location, elev. 650 m, 9 September 2016, N. K. Zeng2906 (FHMU1878); same location, elev. 650 m, 26 July 2017, S. Jiang90 (FHMU4601); Jianfengling of Hainan Tropical Rainforest National Park, elev. 850 m, 6 August 2009, N. K. Zeng481 (FHMU2397); same location, elev. 850 m, 6 August 2009, N. K. Zeng483 (FHMU2398); same location, elev. 850 m, 3 July 2012, N. K. Zeng1038 (FHMU2405); same location, elev. 850 m, 9 July 2013, N. K. Zeng1255 (FHMU813); same location, elev. 850 m, 4 July 2014, N. K. Zeng1579 (FHMU1058); same location, elev. 850 m, 27 June 2018, N. K. Zeng3425 (FHMU3126).

Notes: Our recent collections and the holotype of *C. hygrophoroides* phylogenetically group together with high statistical support are presented in Figure 1. Moreover, the new specimens match well with the protologue of *C. hygrophoroides*, except that the color of the pileal surface is described as “bright red” by Shao et al. (2014) whereas that of our new specimens is distinctly orange-yellow. It is worth noting the color of *Cantharellus* species sometimes is not constant (Buyck et al., 2016c; Olariaga et al., 2017); thus, we suggest these new specimens are *C. hygrophoroides* although the color of the pileal surface between our new collections and the holotype of *C. hygrophoroides* is slightly different.

***Cantharellus laevigatus*** N.K. Zeng, Y.Z. Zhang, Z.H. Chen & W.F. Lin, sp. nov.

Figures 2G,H, 6.

**Figure 6:**
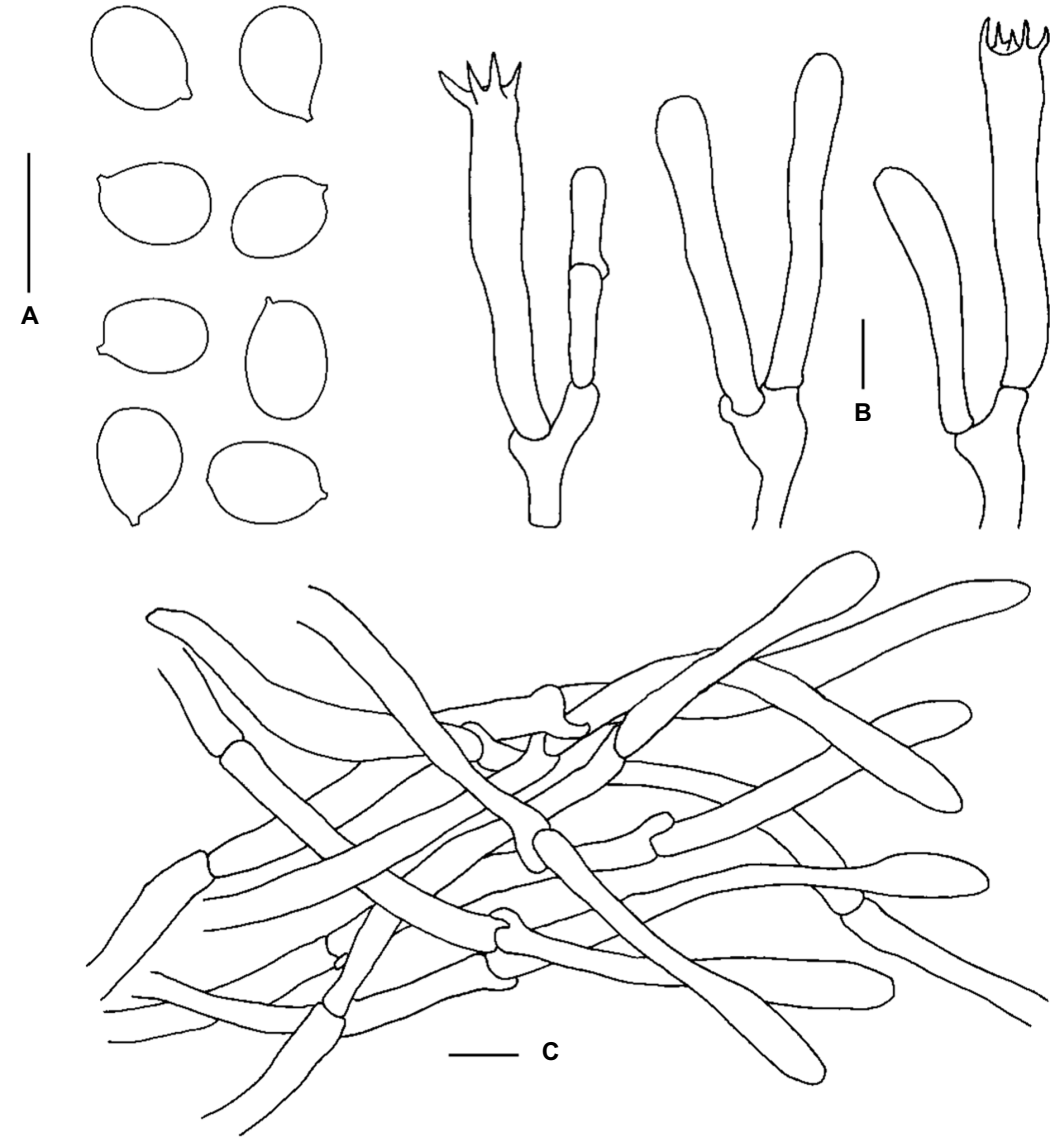
Microscopic features of *Cantharellus laevigatus* (FHMU6951, holotype). **(A)** Basidiospores. **(B)** Basidia. **(C)** Pileipellis. Scale bars = 10 μm. Drawings by Y. Z. Zhang.

MycoBank: MB845019.

Diagnosis: Differs from other species of *Cantharellus* subgen. *Magni* by a small- to medium-sized basidioma, an orange-yellow pileus, a smooth hymenophore, and smaller basidiospores.

Etymology: Latin “*laevigatus*” refers to the smooth hymenophore of the new species.

Holotype: China. Hunan Province: Sangzhi county, Badagong Mountain, Tianping Mountain, elev. 1,478 m, 31 July 2020, Z. H. Chen MHHNU32013 (FHMU6955). GenBank accession number: 28S = ON117824, *TEF1* = ON340615.

**Basidiomata** small- to medium-sized. **Pileus** 2.3–5.2 cm in diameter, plano-convex to infundibuliform, margin slightly incurved, irregular, often wavy and lobed; surface dry, egg-yolk yellow (3A6) to orange-yellow (4A5), shiny; context above stipe about 0.2 cm in thickness, yellowish (4A2), unchanging in color when injured. **Hymenophore** smooth, decurrent, lemon yellow (1A6). **Stipe** 2.3–4.2 × 0.5–0.8 cm, subcylindrical, central, solid, hollow when old; surface dry, white (2A1); context yellowish (3A2). **Taste** and **Odor** not distinctive. **Spore print** not obtained.

**Basidiospores** [120/10/6] 6.5–7.9–8.5(−9) × 5–5.6–6(−7.5) μm, Q = (1.17–)1.23–1.6(−1.64), Q_m_ = 1.42 ± 0.11, broadly ellipsoid to ellipsoid, thin- to slightly thick-walled (up to 0.5 μm), smooth, yellowish in KOH. **Basidia** 42–64 × 6.5–9 μm, narrowly clavate, thin-walled, 4–5–spored, yellowish in KOH; sterigmata 6.5–7 μm in length. **Cystidia** absent. **Pileipellis** a cutis composed of cylindrical, 4.5–11 μm wide, slightly thick-walled (0.5–0.8 μm) hyphae, faintly pale yellow in KOH; terminal cells 45–128 × 5–10 μm, thin- to slightly thick-walled (up to 0.5 μm), subcylindrical to subclavate, with obtuse apex. **Clamp connections** few in all tissues.

Habitat: Scattered or gregarious on the ground in forests dominated by fagaceous trees.

Known distribution: Central and eastern China (Hunan and Zhejiang Provinces).

Additional specimens examined: China. Zhejiang Province: Hangzhou city, Tianmushan Nature Reserve, elev. 1,100 m, 22 July 2020, W. F. Lin4-1, W. F. Lin4-2 (FHMU6951 and FHMU6956). Hunan Province: Sangzhi county, Badagong Mountain, Tianping Mountain, elev. 1,456 m, 31 July 2020, Z. H. Chen MHHNU32011 (FHMU6953); same location, 31 July 2020, Z. H. Chen MHHNU32014, Z. H. Chen MHHNU32061, Z. H. Chen MHHNU32009 (FHMU6952, FHMU6954, and FHMU6950).

Notes: Phylogenetically speaking, *C. laevigatus* is closely related to *C. magnus*, also a species of subgen. *Magni.* Although the value of phylogenetic distance between the two taxa is not high (Figure 1), which was also observed between *C. cibarius* and *C. roseocanus* (Redhead, Norvell & Danell) Redhead, Norvell & Moncalvo (Foltz et al., 2013), *C. magnus* featured by a larger basidioma (pileus up to 20 cm in diameter) and larger basidiospores measuring (8.5–)9–11(−11.5) × (6.5–)6.8–7.5(−8.0) μm (Cao et al., 2021) is morphologically different from *C. laevigatus.* Thus, we proposed the new taxon “*C. laevigatus.*”

In China, *C. laevigatus* was misidentified as *C. hainanensis* N.K. Zeng, Zhi Q. Liang & S. Jiang (Chen and Zhang, 2019), a species also has a smooth hymenophore (An et al., 2017). However, the latter is a member of subgen. *Cantharellus. Cantharellus cibarioides* (Heinem.) Buyck, *C. eccentricus* Buyck, V. Hofst. & Eyssart., *C. flavolateritius* Buyck & V. Hofst., *C. incrassatus* Buyck & V. Hofst., *C. lateritius* (Berk.) Singer, *C. laevihymeninus* T. Cao & H.S. Yuan, *C. sebosus* Buyck, Randrianj. & V. Hofst., *C. sublaevis* Buyck & Eyssart., and *C. vaginatus* S.C. Shao, X.F. Tian & P.G. Liu also have smooth hymenophores (Shao et al., 2011; Buyck, 2014; Buyck et al., 2016c; Cao et al., 2021). However, *C. cibarioides*, *C. sublaevis*, and *C. sebosus* belong to subgen. *Rubrini* Eyssart. & Buyck (Buyck, 2014; Buyck et al., 2014); *C. eccentricus*, *C. flavolateritius*, *C. incrassatus*, *C. laevihymeninus*, *C. lateritius*, and *C. vaginatus* also are members of subgen. *Cantharellus* (Buyck, 2014; Buyck et al., 2014). In addition, *C. neocaledoniensis* Buyck, V. Hofst., Eyssart. & Ducousso and *C. solidus* De Kesel, Yorou & Buyck, two species waiting to be defined in the subgeneric ranking, are characterized by smooth hymenophores either (Buyck, 2014; Buyck et al., 2014). However, *C. neocaledoniensis* has narrower basidiospores measuring (6.2–)6.6–7.33–8(−8.5) × (4.2–)4.5–4.96–5.4(−6) μm and abundant clamp connections in all tissues. It is distributed in New Caledonia, associating with *Melaleuca* L./*Acacia* Mill (Buyck, 2014); *C. solidus* has larger basidiospores measuring (8.3–)8.4–10.2–12(−12.5) × (6.3–)6.6–8.1–9.5(−9.6) μm, two-spored basidia, and it grows under the West African humid gallery forest (De Kesel, 2011; Buyck, 2014).

## Discussion

In the present study, five phylogenetic species of *Cantharellus* were recognized (Figure 1), three lineages were described as new species: *C. bellus*, *C. cineraceus*, and *C. laevigatus*, one was previously described taxon: *C. hygrophoroides*, and the remaining one was not defined due to the paucity of the materials. *Cantharellus bellus* and *C. laevigatus* are both members of subgen. *Magni*, whereas *C. cineraceus* and *C. hygrophoroides* belong to subgen. *Afrocantharellus.* As mentioned earlier, subgen. *Afrocantharellus* has been divided into two sections (Buyck et al., 2014). Our molecular data indicated *C. cineraceus* and *C. hygrophoroides* are members of sect. *Cutirellus* (Figure 1).

In addition to the four described taxa of subgenera *Afrocantharellus* and *Magni*, *C. cerinoalbus* and *C. magnus* were also described/reported in previous studies (Song et al., 2017; Cao et al., 2021). It is worth noting that the Chinese collections labeled as *C. cerinoalbus* and our new species *C. cineraceus* grouped together with high statistical support (Figure 1); moreover, judging from the descriptions of Chinese specimens identified as *C*. *cerinoalbus* (Song et al., 2017), they match well with those of *C. cineraceus*. Thus, we are sure the specimens identified as *C. cerinoalbus* from China are really *C. cineraceus.* Interestingly, the other two new collections (FHMU6948 and FHMU6949) also from the south of China are probably the true *C. cerinoalbus* for they grouped with the isotype of the Malaysian species with statistical support (Figure 1). Unfortunately, due to the paucity of the two materials, they were not studied thoroughly. Thus, the occurrence of *C. cerinoalbus* in China will be confirmed with more collections made and more DNA sequences obtained in future.

Earlier studies indicated the taxa of subgen. *Afrocantharellus* were all described from tropical areas of the world including Africa, Madagascar, and Malaysia (Corner, 1966; Buyck et al., 2014), while species of the subgenus, viz. *C. hygrophoroides,* was uncovered in tropical China (Shao et al., 2014). In the present study, *C. cineraceus* was described from subtropical China, which extends the range of distribution of subgen. *Afrocantharellus.*

It is noteworthy that collections identified as *C. splendens* appear in four parts of the tree; one of them (BB 96.306 and BB 96.199) corresponds to the true *C. splendens* for isotype of the taxon included in the lineage, the second (ADK 6071, JD 896, and JD968) awaits identification, the third (DDT57) seems to be *C. symoensii* Heinem. for the specimen and the epitype of *C. symoensii* group together with statistical support, and the fourth (DDT17) seems to be *C. platyphyllus* Heinem. for the collection clustered with the epitype of *C. platyphyllus* (Figure 1). Moreover, one specimen (DDT63) labeled as “*C. cyanescens* Buyck,” being also clustered with the epitype of *C. platyphyllus*, is most likely to be *C. platyphyllus* (Figure 1).

As already noted by previous studies, *C. cuticulatus* and *C. splendens* have trichodermal structures in the pileipellis (Corner, 1966; Buyck et al., 2014), and *C. hygrophoroides* were also observed to have intricate trichodermal pileipellis. In addition, the Chinese species classified in the subgen. *Afrocantharellus* lack bluish context, which was observed on African *C. platyphyllus* and its Malagasy subspecies *bojeriensis* (Buyck et al., 2014).

We also noted that most species of *Cantharellus* with smooth hymenophores belong to subgen. *Cantharellus* and subgen. *Rubrinus*, respectively (Shao et al., 2011; Buyck, 2014; Buyck et al., 2016c; An et al., 2017). The recently erected subgen. *Magni* was also reported to have a smooth hymenophore (Cao et al., 2021), and our new species *C. laevigatus* is the second species of the subgenus uncovered with a smooth hymenophore. Interestingly, a well-developed hymenophore was observed from our new species *C. bellus*, also a member of subgen. *Magni*, which indicates the diagnostic features of subgen. *Magni* should be revised.

## Data availability statement

The datasets presented in this study can be found in online repositories. The names of the repository/repositories and accession number(s) can be found in the article/Supplementary material. The data presented in the study are deposited in the Genbank and MycoBank repository, Genbank accession number: ON089297–ON089298, ON102890–ON102902, ON117818–ON117821, ON117823–ON117825 (28S); ON191964–ON191968, ON202824, ON237706–ON237708, OP251152–OP251153, ON340611–ON340616, ON340619 (TEF1); MycoBank accession number: MB845017–MB845019.

## Author contributions

Z-QL and N-KZ contributed to the conceptualization. Y-ZZ performed the methodology and conducted the formal analysis. The original draft preparation was written by Y-ZZ and H-ZQ. The experiment was performed by Y-ZZ. N-KZ, Z-HC, and W-FL. SJ carried out the resources. N-KZ and Z-QL wrote, reviewed, and edited the manuscript and directed the data. N-KZ was responsible for project management and funding access. All authors contributed to the article and approved the submitted version.

## Funding

This study, including funds for open access publication fees, was supported by the National Natural Science Foundation of China (No. 32160001).

## Conflict of interest

The authors declare that the research was conducted in the absence of any commercial or financial relationships that could be construed as a potential conflict of interest.

## Publisher’s note

All claims expressed in this article are solely those of the authors and do not necessarily represent those of their affiliated organizations, or those of the publisher, the editors and the reviewers. Any product that may be evaluated in this article, or claim that may be made by its manufacturer, is not guaranteed or endorsed by the publisher.

## Footnotes

^1^ https://www.ncbi.nlm.nih.gov/genbank

^2^ http://tree.bio.ed.ac.uk/software/tracer/
